# Jelly-Z: swimming performance and analysis of twisted and coiled polymer (TCP) actuated jellyfish soft robot

**DOI:** 10.1038/s41598-023-37611-1

**Published:** 2023-07-08

**Authors:** Pawandeep Singh Matharu, Pengyao Gong, Koti Pramod Reddy Guntaka, Yara Almubarak, Yaqing Jin, Yonas T. Tadesse

**Affiliations:** 1 Humanoid, Biorobotics and Smart Systems Laboratory (HBS Lab), Erik Jonsson School of Engineering and Computer Science, The University of Texas at Dallas, Richardson, TX 75080 USA; 2 Fluids, Turbulence Control and Renewable Energy Laboratory, Department of Mechanical Engineering, The University of Texas at Dallas, Richardson, TX 75080 USA; 3 SoRobotics Laboratory, Department of Mechanical Engineering, Wayne State University, Detroit, MI 48202 USA

**Keywords:** Mechanical engineering, Soft materials

## Abstract

Monitoring, sensing, and exploration of over 70% of the Earth’s surface that is covered with water is permitted through the deployment of underwater bioinspired robots without affecting the natural habitat. To create a soft robot actuated with soft polymeric actuators, this paper describes the development of a lightweight jellyfish-inspired swimming robot, which achieves a maximum vertical swimming speed of 7.3 mm/s (0.05 body length/s) and is characterized by a simple design. The robot, named Jelly-Z, utilizes a contraction–expansion mechanism for swimming similar to the motion of a Moon jellyfish. The objective of this paper is to understand the behavior of soft silicone structure actuated by novel self-coiled polymer muscles in an underwater environment by varying stimuli and investigate the associated vortex for swimming like a jellyfish. To better understand the characteristics of this motion, simplified Fluid–structure simulation, and particle image velocimetry (PIV) tests were conducted to study the wake structure from the robot’s bell margin. The thrust generated by the robot was also characterized with a force sensor to ascertain the force and cost of transport (COT) at different input currents. Jelly-Z is the first robot that utilized twisted and coiled polymer fishing line (TCP_FL_) actuators for articulation of the bell and showed successful swimming operations. Here, a thorough investigation on swimming characteristics in an underwater setting is presented theoretically and experimentally. We found swimming metrics of the robot are comparable with other jellyfish-inspired robots that have utilized different actuation mechanisms, but the actuators used here are scalable and can be made in-house relatively easily, hence paving way for further advancements into the use of these actuators.

## Introduction

The design and development stages of soft robots incorporate several variables such as flexibility, control, size, weight, power, and cost. To achieve that, the recent focus has been on combining soft passive materials as a flexible dynamic structure, and novel smart material for actuation, sensing, and control. Doing so serves as the steppingstone for the next generation of biomimetic soft robots making them not only smarter with the integration of sensors but also highly compliant. With these types of soft robots many challenges arise such as stiffness control^1,2^ and stiffness modulation.

The field of underwater robotics has become highly important for both military and industrial applications. Divers and traditional ROVs are faced with difficulties while operating in inhospitable environments that expose them to extreme temperatures and pressure. As a result, current solutions are equipped with expensive, and heavy protective equipment which can make underwater operations even harder to achieve. Adaptability is key in dealing with these obstacles that are presented in harsh and yet to be explored aquatic spaces. Researchers have already taken advantage of this by deploying a soft robot in the Mariana trench (depth 11,034 m underwater)^3^. The focus on underwater biomimetic robots can be seen in many robotic structures with the success of using synthetic material to present a high degree of freedom robot such as a 3D printed musculoskeletal joint with variable stiffness^4,5^, robotic fish^6–9^, octopus like tentacles^10–13^ and jellyfish like robots^14,15^. Information on kinematics of real jellyfish is provided through studying the hydrodynamic interactions during swimming of a medusae on vortex formation and thrust production based on particle image velocimetry (PIV) methods^16^. Recently, biomimetic robot producing Nautilus-like thrusts have been 3D printed, computed from hydrostatic simulations^17^. Matharu et al.^18^ recently provided a few guidelines for designing jellyfish soft robots based upon the investigation conducted on real jellyfish animals and based on the review conducted by Costello et al.^19^ It is stated that one of the key parameters of jellyfish swimming is that its contraction cycle must be close to or less than 50% of the relaxation cycle (duty cycle ~ 50%).

This paper focuses on the analysis of the swimming performance of a soft jellyfish inspired robot (Jelly-Z) published in IMEKO ACTA^20^ using novel material for actuation, twisted and coiled fishing line thread incorporated with nichrome wire (${\mathrm{TCP}}_{\mathrm{FL}}$). In general, TCPs are soft polymer materials that are initially fully twisted causing them to coil in a spring like shape. They contract when heated, depending on their construction. The stimuli can be activated by Joule heating or convection heating such as using fluids with varying temperature or hot and cold air. The Jelly-Z prototype specifications are explained in Table 1. The natural jellyfish has the capability of swimming long distances while exerting low energy for propulsion. Attempts to integrate artificial muscles into a jellyfish like and other soft robotic structures have been made such as using shape memory alloys (SMA)^21–23^, twisted and coiled polymers (TCP) using silver-coated nylon^24^, dielectric elastomers (DE)^25–28^, pneumatics^29^, ionic polymer metal composites (IPMC)^30^, and by hydrogen fuel^31^.

**Table 1:** Jelly-Z prototype specification.

Property	Specification
Dimensions	Ф150mm × 5 mm (not pre-tensioned) Ф150mm × 33 mm (pre-tensioned with TCP_FL_)
Mass	168 g
Speed at 2.4 A current	7.3 mm/s
Actuation frequency	0.33 Hz at max
Actuators	Twisted and coiled polymer fishing line (TCP_FL_) with nichrome
Power (Current and Voltage)	134 W (2.4 A and 56 V)

The scientific objective of this study is understanding the behavior of soft silicone materials actuated by novel self-coiled TCP_FL_ in an underwater environment by varying stimuli and investigate the associated vortex for swimming like a jellyfish. The silicone is a hyperelastic material and the polymer actuators are semi crystalline materials. The coupled system will be another nonlinear system, and when these materials are dynamically actuated in water, the resulting structure is a complex dynamic system. We studied this system and tried to understand and quantify them in both experimental and theoretical framework. The stimuli or input was varied by changing either the current magnitude or actuation frequency, to observe the dynamic system. We looked at different regions to approximate with linear trend for instance the actuation properties of the polymer muscles.

To understand the hydrodynamics of the natural jellyfish swimming, pioneers studied the flow field through flow visualization technologies such as dye visualization^32–34^ and particle image velocimetry (PIV)^35–38^. They found 3 key aspects of thrust generation mechanism^19^. Specifically, they showed that a suction force is generated during the contraction of the bell that drives the whole body moving forward^35^. During relaxation, water is drawn into the bell, which is highly modulated by the vortex generated in this phase that provides the pressure difference to push the bell further forward^39^. Despite the pressure driven process, the vortex produced via previous cycle acts as a virtual wall that enhances the propulsive performance of the jellyfish^40^, which is similar to the ground effect found in fish swimming near the sea surface^41^ or airfoil flapping near the solid substrate^42^. Thus, to test the performance of Jelly-Z, PIV and propulsive force measurement are adopted.

Not many successful swimming robots are known to explicitly leverage the swimming technique and morphology of jellyfish as found in nature while having it exclusively actuated with twisted and coiled polymer fishing lines (${\mathrm{TCP}}_{\mathrm{FL}}$). This actuation method avoids the usage of rigid electronic components like motors or pumps for actuation, rather it utilizes a totally soft actuation technique that eliminates the complexity of adding mechanical components and can be deployed safely in sensitive crowded environments which is a precursor towards developing soft robots for commercial use in future. Pneumatic artificial muscles and electric motors have some drawbacks for this application as they are bulky, noisy, and expensive while the electric motors have limitations of size, space, weight, and functionality while submerged in an underwater environment. Moreover, the electric motors do not adhere to the bioinspired design methodology.

Shape memory alloy (SMA) wires and dielectric elastomeric actuators (DE) are two common actuators that have been used in jellyfish-like robots as shown in Supplementary Table S1. Linear shaped SMA wires and coiled shaped require high input power (~ 3 times than in air)^21^ when used in an underwater setting. Moreover, they are difficult to control due to their hysteresis properties. Another important reason for choosing TCP_FL_ over similar linear actuators such as shape memory alloys (SMA) is that the material used to fabricate these muscles are polymers, which are eco-friendly, lightweight, and inexpensive. DEs need very high input voltage to operate^27,43^ which is dangerous specifically in an underwater setting. The fabrication of TCP_FL_ is tunable by simply changing the precursor fiber’s or the nichrome wire’s geometry. The working mechanism of TCP_FL_ artificial muscles is similar to a biological muscle, hence we chose this actuator.

In this work, a biomimetic robot, Jelly-Z, is described which leverages the swimming principle employed by a jellyfish for underwater locomotion with a cyclic contraction–expansion mechanism generating thrust force in upwards direction for net forward motion. For quantifying the utility of the robot for practical underwater robotic applications, its swimming performance is characterized and controlled by varying input current supplied to the TCP_FL_ actuators resulting in varying swimming speeds. Understanding the performance of TCP_FL_ actuators is important for various conditions to utilize the most optimum parameters for swimming applications. This paper demonstrates (1) a simple jellyfish inspired underwater swimming robotic structure and its swimming performance critically, (2) control of swimming speed with varying input current delivered to the TCP_FL_ actuators and swimming performance comparison with other jellyfish robots, (3) modeling, validation and understanding the actuation behavior of the TCP_FL_ actuators in underwater environment, (4) study of flow dynamics, thrust production, and thrust efficiency of Jelly-Z at 0.33 Hz, in the same range of actuation frequency of real jellyfish (0.1 Hz to 1 Hz)^44–47^, to validate the locomotion effectiveness experimentally.

## Results

**Figure 1:**
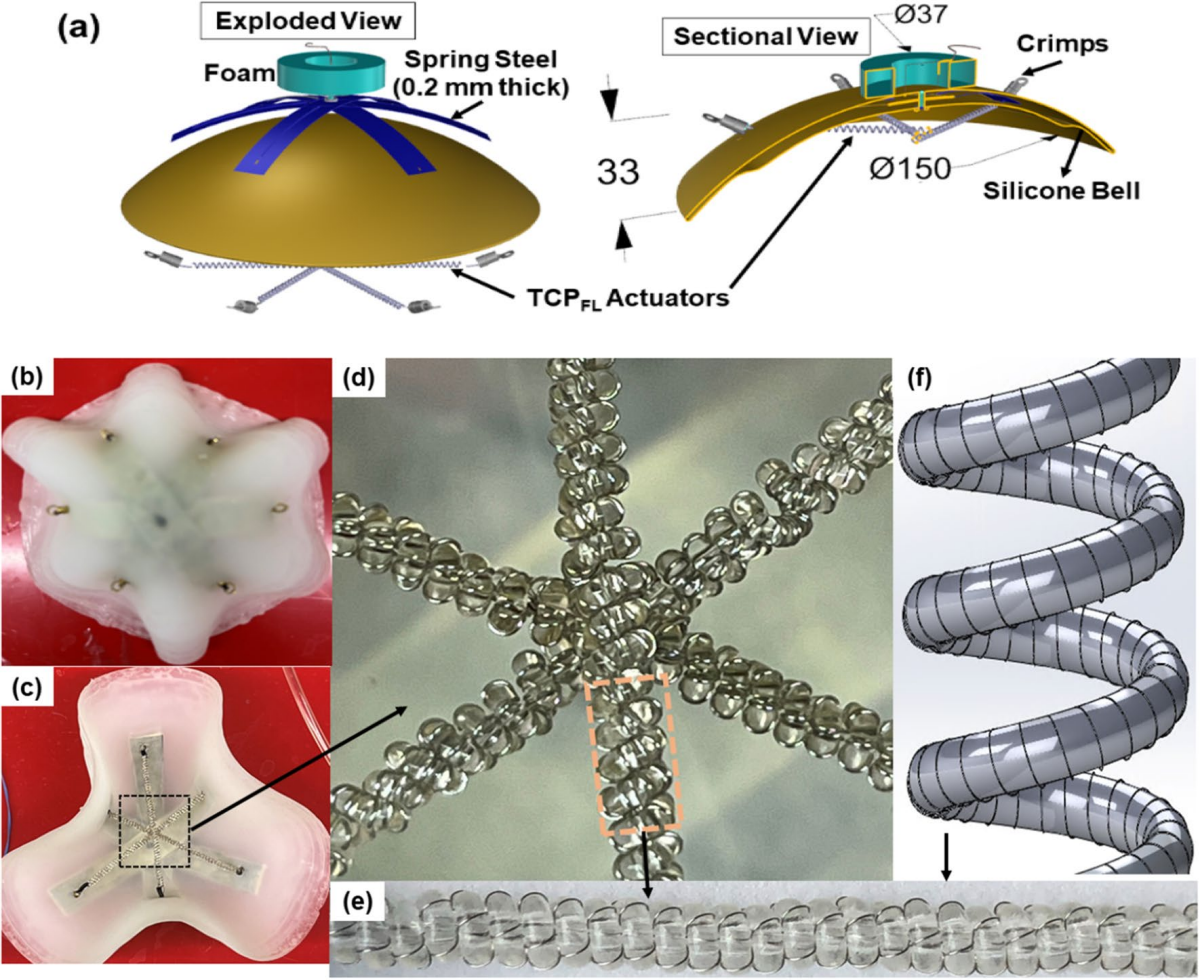
The overview of Jelly-Z prototype. (**a**) 3D CAD model (exploded and sectional views) of the robot, (**b**) Top view of Jelly-Z robot, (**c**) Bottom view of Jelly-Z robot, (**d**) Internal Assembly of TCP_FL_ actuators, (**e**) Zoomed-in view of the actuators, (**f**) CAD model of TCP_FL_ actuators.

We started by conceptualizing Jelly-Z to have a compact modular and soft structure. After the CAD design was completed, we fabricated the robot using 3D printed mold for the silicone. The assembly was built to create the final structure (bell shape, artificial muscles, spring steels) with all its electrical wiring in the bell margin (Fig. 1a–f). The robot weighs 168 g with all its components and measures ф150 mm X 5 mm including a 20 mm passive skirt all around. Electrical power is provided through the tether of thin insulated wires from outside the water, while the robot prototype is operated underwater. The mass of all components was distributed symmetrically across the robot with minimal weight throughout the robot structure aiding in faster swimming speeds for a jellyfish robot of such size. To offset the weight, a foam ring was integrated at the top of the bell center, until the robot was stable and neutrally buoyant.

### Experimental characterization with predictive model of TCP_FL_ actuators

Testing of the custom-made novel actuator is essential to understanding the performance of it working in various scenarios. The basic questions are: What is the behavior of the TCP actuator when it is functioning in an underwater environment? What are the parameters that affect the performance of the TCP actuator? These are very limited studies that are available when it comes to the TCP actuated in an underwater setting for robotic application^20,48^. As a result, an experimental characterization was performed on the self-coiled TCP_FL_ actuator, in which the parameters can be noted in Table 2. The testing set up and the experimental conditions are explained in the Methods section*.* A constant load of 500 g was applied as a pre-stress parameter to the actuator because the maximum stroke is achieved at that load^5,48,49^.

**Table 2:** Mechanical and electrical properties of TCP_FL_ in water.

Material	Nylon (6,6)
Type of actuation	Electrothermal
Type of resistance wire	Nichrome (Ni/Cr)
Fishing line diameter (mm)	0.8
Nichrome diameter (µm)	160
Length of the actuator after coiling (mm)	52, 55, 58
Diameter of the actuator after coiling(mm)	2.1
Annealing temperature (°C) /Time (min)	180/90
Mass of the actuator (kg)	0.4 × 10^–3^
Resistance (Ω)	52, 55, 58
Heating time/Cooling time (s)	5/ 25
Current (A)	0.45–0.75
Voltage (V)/Power (W)	56/36.6
Duty cycle (%)/Actuation frequency tested (Hz)	16.6/0.033
Blocking force (experimental)	8N

**Figure 2:**
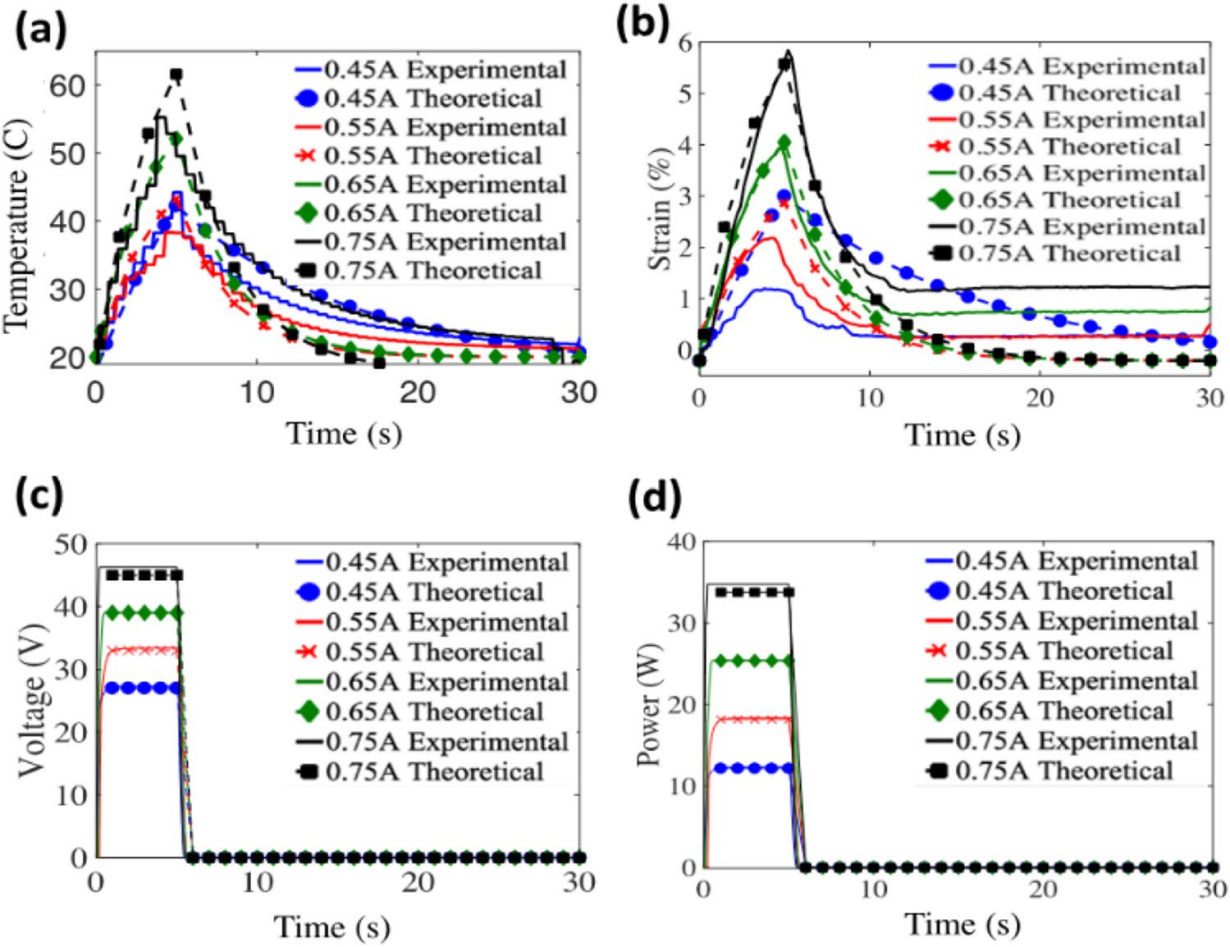
Comparison of isotonic testing at 500 g loading conditions for a single TCP_FL_ in an underwater environment with predicted model. (**a**) Experimental results of temperature vs simulated temperature from the model, (**b**) Experimental results of strain vs. simulated strain from the model, (**c**), (**d**) Experimental results of Voltage, Power vs simulated results from the model. The values of “h” at different input currents are 1529 W/(m^2^K) for 0.45A, 1580 W/(m^2^K) for 0.55A, 1603 W/(m^2^K) for 0.65A and 1550 W/(m^2^K) for 0.75A. Note: The hysteresis plots for heating cycle, cooling cycle and theoretical results can be seen in Supplementary Fig. S3.

Figure 2a–d show the results for output temperature, strain, output voltage, and input power respectively. The maximum actuation strain of 6% is noted for an input current of 0.75 A while the highest temperature reached is ~ 62 °C. At lower input currents of 0.65 A, 0.55 A and 0.45 A, actuation strain/displacement reduces due to the decrease in heat input. This can be seen from Fig. 2a, where the temperature of the TCP_FL_ shows a decreasing trend. Power measurements are conducted based on the equation P = VI, where P is input power, V is input voltage measured from NI DAQ 9221, and I is input current. At input currents of 0.45 A, 0.55 A, 0.65 A, 0.75 A (Fig. 2a), the voltages measured from NI DAQ 9221 module were 27 V, 33 V, 39 V, 47 V respectively. This shows that as the input current to the TCP_FL_ artificial muscles increases, the input power required to actuate the TCP_FL_ artificial muscles also increases. This suggests that the water is taking the heat away as the temperature should have reached up to 100 °C to get higher actuation strain. The variation of input current and heating time is very critical due to the temperature sensitivity of the actuator. If the temperature exceeds its melting point (250 °C) when heat is applied, the muscle will burn and will no longer actuate. The highest input current tested was 0.75A as the TCP_FL_ actuator got damaged within the first 5 cycles, when a higher input current (0.85A) is provided. Supplementary Fig. S3 illustrates the relationship between experimental and simulation readings for temperature and strain for self-coiled TCP_FL_ in water.

Equation (1) can be used to predict the heat rise in the TCP_FL_ actuator which consists of input power provided to the actuator and the heat loss due to the convective heat transfer.

1 $$m{C}_{p}\dot{T}={i}^{2}R-hA\left(T\left(t\right)-{T}_{\infty }\right).$$

Rewriting Eq. (1) and rearranging, the temperature T(t) is expressed as in Eq. (2).

2 $$T\left(t\right)= \frac{-m{C}_{p}\dot{T}+{i}^{2}R}{hA}+ {T}_{\infty },$$

where *m* is mass, *Cp* is the specific heat capacity (Cp of the fishing line material = 1700), *i* is the input current, *R* is the resistance of the actuator, *h* is the convective heat transfer coefficient,* A* is the surface area of the actuator, and ${T}_{\infty }$ is the ambient temperature (temperature of the surrounding environment) and $t$ is the time. A linear relationship also exists between temperature and strain. Equation (4) was formulated using linear regression-based formula to predict the strain ($S$) of the TCP_FL_. The convective heat transfer coefficient $h$ is obtained by rearranging the term in Eq. (1) and can be written as Eq. (3). This value can be obtained experimentally from one data set since we have the time dependent temperature rate and temperature as well as the input ${i}^{2}R$, and material properties.

3 $$h= \frac{m{C}_{p}\dot{T} - {i}^{2}R}{A(T- {T}_{\infty })}.$$

The resistance R and the heat transfer coefficient h were obtained from a few experimental data and used for other model predictions.

4 $$S(t)= \gamma T(t)+\beta$$

where γ is the slope, and β is the intercept. The temperature and strain predicted using Eqs. (2) and (4) are plotted alongside the experimental results in Fig. 2. The values of γ, β are 0.138 and 2.86 respectively which were extracted from experimental results. This simplistic modeling allows us to predict how the overall system will behave under various material and environmental changes without the need for experimental validation.

In the developed model, we assume the value of “h” is constant for each current according to Eq. (3) presented in the paper. The model runs for several iterations and the value of h updates after each iteration based on the previous predicted temperature point “T”. $i$ varies according to the heating and cooling cycles. While values such as m, ${C}_{p}$, A, $R$, and ${T}_{\infty }$ are constant throughout the iteration process for each individual current. The values of “h” at different input currents are 1529 W/(m^2^K) for 0.45A, 1580 W/(m^2^K) for 0.55A, 1603 W/(m^2^K) for 0.65A and 1550 W/(m^2^K) for 0.75A. The linear approximation that we considered for theoretical study is based on the heating cycle only and does not consider the cooling cycle. Hence, the values of R^2^ (Fig. S3b) for the cooling cycle are from 0.67 to 0.87. But the heating cycle regression coefficient is between 0.92 and 0.99, which indicates linear approximation is valid in this region.

### Vertical swimming tests at different input currents

**Figure 3:**
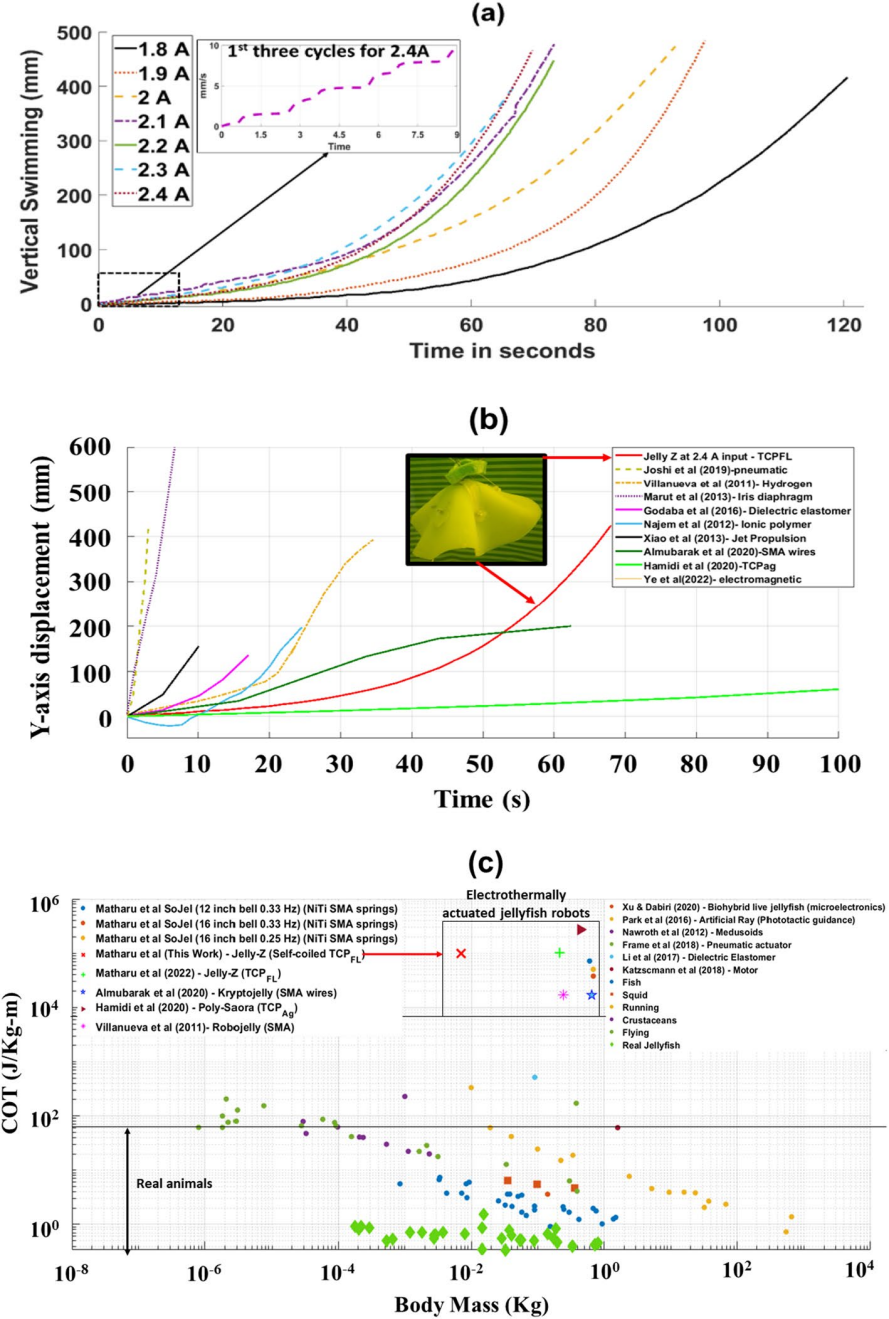
(**a**) Swimming speeds measured in benchtop test. The swimming speed of the biologically inspired robot prototype is shown for varying input currents. (**b**) Vertical swimming speeds of Jelly-Z prototype at 2.4 A compared with other jellyfish-inspired prototypes in literature whose swimming data is available in publications^14,21,22,24,27,50–53^. (**c**) Net cost of transport relative to body mass for Jelly-Z (This work) prototype, and for swimming, flying, and running animals^19^, along with other major underwater robots bioinspired by different animals and also using different actuators^8,18,20,22,24^.

We conducted 7 swimming experiments on the robot prototype at different input currents to control its vertical swimming performance by increasing the input power. The robot was made neutrally buoyant in a 70-gallon fishing tank having dimensions, 920 mm × 585 mm × 460 mm. Figure 3a shows that with the increase in input current, the swimming speed also increases as the actuation displacement of the three, 55 mm long TCP_FL_ actuators increase along with input current. The robot takes 21 actuation cycles to swim a vertical distance of 460 mm in 63 s at an input current of 2.4 A (56 V), while it swims a similar distance in 120 s at an input current of 1.8 A (52 V). The actuation cycles were 0.33 Hz frequency and 50% duty cycle, which corresponds to 1.5 s heating (contraction cycle) and 1.5 of cooling (relaxation cycle—0 A). It is observed for all the seven experiments that the robot is slower in the first 10–15 cycles, while it steadily gains momentum and increases speed as it reaches a vertical distance of about 100 mm moving towards the surface of water. This is because the gained momentum aids the robot in accelerating while swimming vertically upwards. Jelly-Z was tested at 2.5A input current, however after 8 to 10 actuation cycles, the integrated TCP_FL_ broke, having a negative impact rather than more actuation displacement.

Figure 3b demonstrates a comparison of Jelly-Z with other well-known jellyfish soft robots. It can be seen that Jelly-Z swims at an average vertical swimming speed of 7.3 mm/s at 0.33 Hz (1.5 s ON–1.5 s OFF), one of the slower speeds amongst contemporary soft jellyfish robots. However, the major benefits of such a soft jellyfish robot is that it is actuated with thermally operated TCP_FL_ artificial muscles which are quiet in operation and provide stealth actuation, which can be beneficial for many underwater exploration and stealth activities. A common metric cost of transport (*COT*) (Eq. (5)) is used for quantifying the effectiveness of biological locomotion techniques. The speed and *COT* of soft robots is generally less than real animals. The *COT* of Jelly-Z is higher than the real animals (Fig. 3c), due to high power consumption, low robot mass and slow swimming speed. Hence, to improve this aspect, we can introduce nanomaterial coating of TCP_FL_ artificial muscles, similar to Piao and Suk^58^ in order to improve interfacial thermal contacts, enhancing both the cooling time of the actuator (improving cycle frequency) and consuming less power. We can also make larger passive flaps and use mandrel-coiled actuators (having higher actuation stroke) as shown by Wu et al.^5^ However, COT is not the only parameter we are concerned, but we also considered the audio noise generated by a particular actuation technology. The audio noise for smart materials such as TCPs is minimal compared to servo motor-based actuators.

### Experiment on thrust force measurement

**Figure 4:**
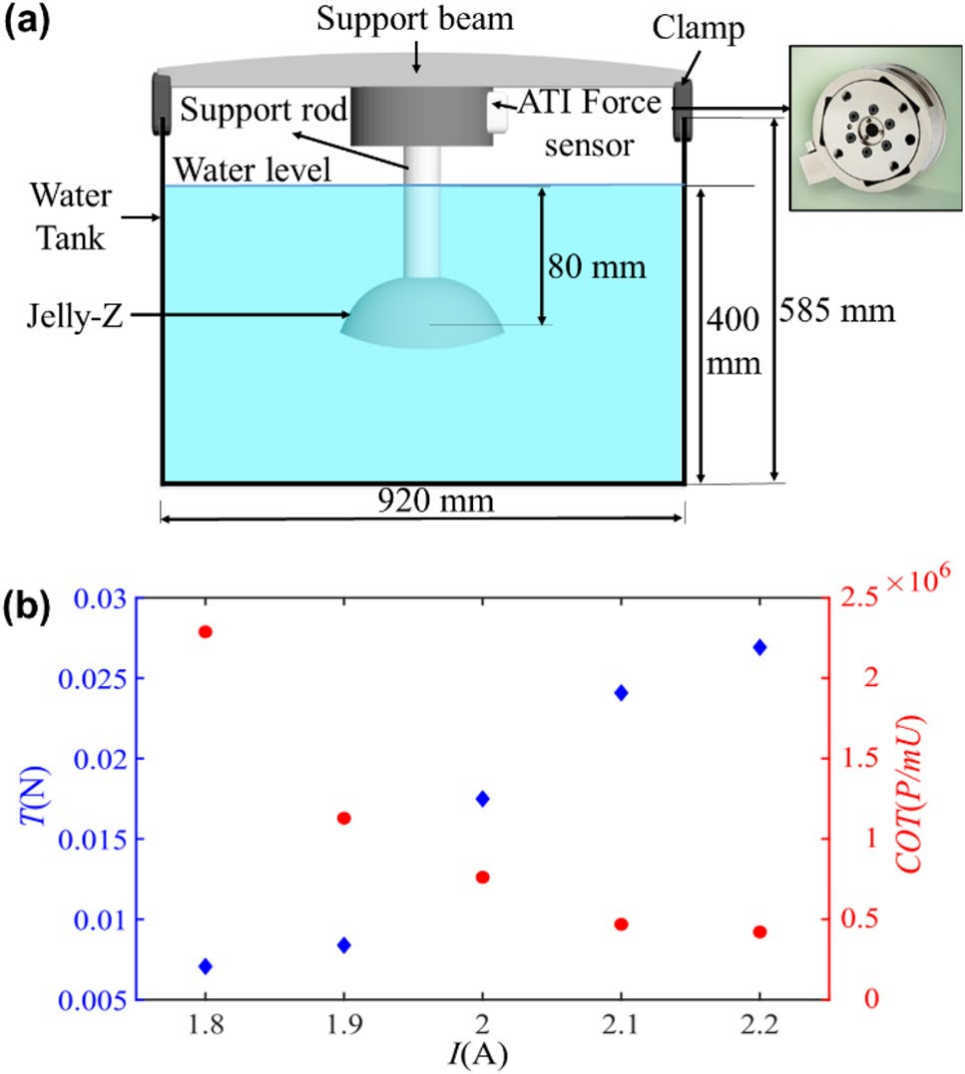
(**a**) Experimental set up of propulsive force measurement; (**b**) left—cycle averaged force across various input currents; right -Cost of transport (COT) across various input currents.

For force measurement, the Jelly-Z prototype was connected to a high-resolution ATI force sensor sampling at a frequency of 1 kHz with an uncertainty less than 1.2% to obtain averaged propulsive force as shown in Fig. 4a. To actuate the silicone bell for various actuation amplitudes in force measurement, the robot is supplied with input currents from 1.8A to 2.2A. It can be seen in Fig. 4b that the magnitude of force exerted by the jellyfish robot increases with increasing input current, which shows a similar trend that can be seen in the swimming experiment. The actuation duration was set to 3 s per cycle with 1.5 s ON and 1.5 s OFF. To further investigate the thrust efficiency of Jelly-Z, the cost of transport (*COT*) is defined by the ratio of input power over output power^59^,

5 $$COT=\frac{P}{mU}$$

where *U* is the corresponding average swimming velocity, the electricity charging power is noted by *P*, computed by the charging voltage *V* and current *I*. This propulsive cost is summarized in Fig. 4b as the function of input current. Specifically, propulsive efficiency increases as power input increases, the highest efficiency is stabilized over 2.1A, while the averaged force for one cycle in Fig. 4b shows a continuously increasing trend. This stabilization of COT is possibly related to energy loss of heat to the surroundings. The COT values for all the cases are similar to previously developed jellyfish robots like Kryptojelly and Polysaora^21,24^.

### Experiment for wake flow dynamics

**Figure 5:**
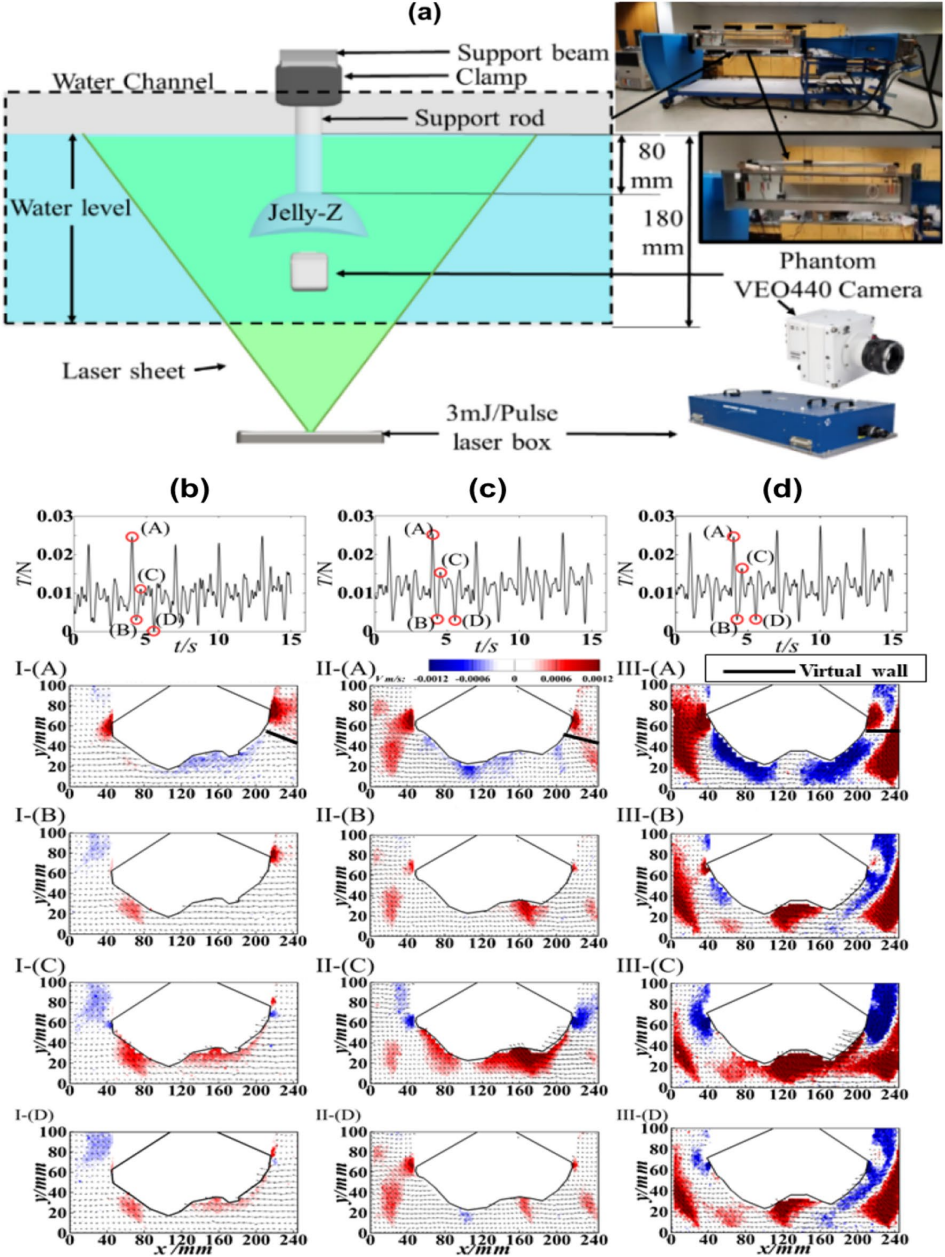
PIV experimental results for the wake flow dynamics of Jelly-Z prototype. (**a**) Experimental setup schematic, (**b–d**) Time series plots and Instantaneous snapshots of velocity contours overlapped with velocity vectors for one cycle of actuation under input currents from 1.8A, 2.0A and 2.2A marked by (I) to (III) respectively. (**A**)’s represents highest velocity repelled momentum underneath the bell regarding the maximum vertical force during contraction; (**B**) is the instant when the contraction ends, and the relaxation starts; (**C**)’s shows the maximum suction momentum instant during relaxation; and (**D**)’s refers to the instant when the relaxation ends, and the next cycle starts. The experiments were conducted at 0.33 Hz actuation frequency (50% Duty cycle).

Representative snapshots of flow surrounding the Jelly-Z prototype and the corresponding thrust force time series of five cycles are illustrated in Fig. 5b–d. For each actuation amplitude at different values of input currents, instantaneous vertical velocity snapshots superimposed by velocity vectors are presented. The thrust force time series for each chosen input current (1.8A, 2.0A, 2.2A) are marked by (I), (II) and (III) respectively. For each chosen case, representative snapshots are marked by (A) to (D) and explained in Fig. 5.

We study the vertical velocity to elucidate clues of pressure distribution in vertical direction. Overall, the vortex-bell structure interaction mechanism is similar for each amplitude. Specifically, a counterclockwise vortex is created at the right-side bell margin during contraction as shown in Fig. 5b–d (I-III, A), which links to the upward high velocity near the front face where a low-pressure region is created. While, beneath the bell, dispelled water generation of this phase is highly modulated by the pressure difference across the bell vertical direction. Higher momentum of dispelled water was created by giving higher flapping amplitude. What’s more, the vortex generated at the bell margin interacts with another vortex underneath the bell from previous cycle, which creates a Virtual Wall effect that enhances the propulsion due to the resistance between these two vortices^60^, this wall effect region is marked as the black line in Fig. 5. The corresponding force measurement shows a peak value of thrust force during the 1.5 s contraction, this peak value locates approximately at 1.0 s. After around 0.25 s, thrust force reaches a local minimum, which links to Fig. 5b–d (I-III, B) where contraction ends, bell starts to relax, and vertical flow velocity fades. For relaxation phase, water is pumped into the bell, which links to a clockwise vortex on the right bell margin instead, pushing the inside face of the robot bell to further move forward. Specifically, peak force generated in this phase is relatively lower than the contraction phase. This high vertical flow momentum is correlated to the local peak of the thrust generation as shown in Fig. 5b–d (I-III, C). As the relaxation fades, another lower local thrust minimum as well as a lower wake flow momentum is generated before steps into another cycle (Fig. 5b–d (I-III, D)). In general, this whole procedure of thrust generation is highly in accordance with the real jellyfish swimming^19^. The purpose of conducting the PIV tests was not to achieve the maximum wake flow, but rather to investigate the wake flow structure with increasing input current. This is the reason why the current range of 1.8A–2.2A was chosen, which is similar to the input currents chosen for the actual swimming tests, rather than conducting a test at maximum input current of 2.4A.

### Fluid–structure feedback

**Figure 6:**
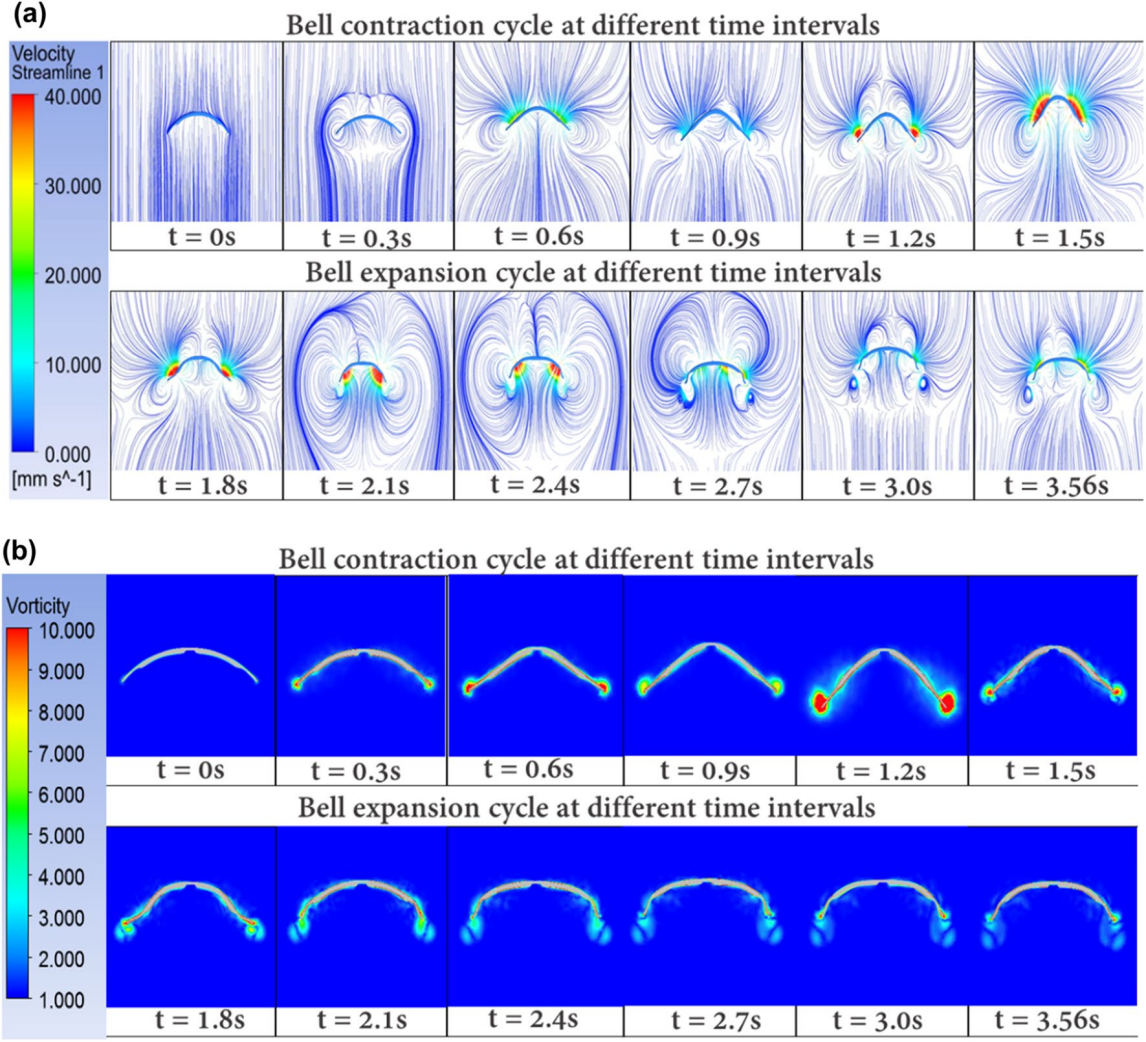
(**a**) Velocity streamline and (**b**) Vorticity contours generated around the robot due to the contraction and expansion motion of the soft structure. The fluid velocity is set as 7.3 mm/s inlet vertical flow.

A simplified fluid–structure interaction simulation is conducted to study the development of the fluid flow around the jellyfish structure due to its bell geometry and bell actuation motion. Results such as the velocity streamline in the downstream and the vortex contours (Fig. 6a,b) direction are extracted. This set up can be used to simulate various design iterations for underwater robots and how the flow may affect the vortices around Jelly-Z’s geometry and its overall swimming performance. The simulation set up and applied boundary conditions are explained in detail in the Methods section and provided Supplementary Material. Each snip is taken at a time step of ~ 0.3 s. and the first 6 snapshots demonstrate the contraction cycle while the remaining 6 demonstrate the relaxation cycle. It is important to note that compared to the experimental work, the simulation assumed a fixed body boundary condition for the jellyfish structure and a velocity equivalent to the jellyfish swimming (7.3 mm/s) was provided as an input to the surrounding fluid (flow input top and flow output bottom). Therefore, we can see that the maximum velocity obtained is ~ 40 mm/s which is observed to be close to the bell tip. This is higher than the velocities measured in the wake region from experimental results, but the nature of the streamline and contour plot presented is according to what we observe physically in Fig. 5. From these results, we can see how the contraction cycle tends to pull the flow inward which creates a high velocity profile within that area. As the bell starts to relax (t > 1.5 s), we can observe that it starts to create larger circulation around the jellyfish bell and then starts to dissipate with smaller circulation by the end of the cycle as shown at t = 3.56 s. The flapping motion generated by the actual jellyfish involves curling of the flaps inwards which induces a certain amount of asymmetry in the wake region and gives them a propulsive advantage as presented in Gemmell et al.^39^. However, the flapping motion implemented in this model is not strong enough to generate any visible curling. In general, the higher velocities shown in simulation can be attributed to two reasons. The first one is the simulation has a velocity of 7.3 mm/s impinging on the jellyfish while in experiment it was in static water. The second one is the maximum velocity in simulation was observed to be close to the bell tip, while in experiment we only measured the wake flow region.

## Discussion

This article introduced and described a biomimetic jellyfish robot (Fig. 1) that uses TCP_FL_ actuators to swim underwater as shown in Fig. 3a. This is the first time that TCP_FL_ actuators (quiet in operation and stealth in actuation) have been utilized and investigated in a jellyfish-inspired robot, making the work novel. Figure 2a–d demonstrates the isotonic testing at 500 g loading conditions for a single TCP_FL_ in an underwater environment with predicted model. The predictive model based on Joule heating shows good fitting with the experimental measurements and can be utilized for estimation for closed loop PID control of TCP_FL_ actuators for various applications requiring different quantities of actuation displacement. This will save excess energy provided as input current and will help to optimize the TCP_FL_ actuation. It will aid to predict the temperature rise and accompanying actuation strain of the TCP_FL_ actuator in underwater conditions and helps in ascertaining the heat loss for a certain input current.

In addition, the robot is fully soft except for the inclusion of very thin (0.2 mm thick) spring steels to aid in the expansion stroke of the TCP_FL_ actuators. The robot prototype is created by casting Ecoflex 00–10 silicone with embedded spring steels and TCP_FL_ actuators making the assembly and disassembly easy. TCP_FL_ actuators were chosen over similar linear actuators such as SMA wires or 6-ply TCPs used in Hamidi et al.^24^ because of higher actuation stroke per cycle, the ease of fabrication, and low material cost of the actuator. Though, high stroke does not necessarily mean faster swimming robot as can be seen in Fig. 3b, the dependence of swimming speed between different robots depends on the size of the passive flap of the silicone bell and the actuation technology. These actuators can be used for quiet underwater operations at various depths where fast swimming is not required. For example, the robots in Fig. 4b that show a very linear vertical profile are actuated by pneumatic actuators as seen in the work presented by Joshi et. al.^50^ which can be used for an application requiring the robot to swim faster in water. These TCP actuators in Hamidi et al.^24^ are different than the TCP presented in this work (TCP_FL_) since the former are made out of silver coated nylon (TCP_Ag_), whereas the latter is using fishing line with nichrome heater wire. It is also different from the actuators presented by Piao and Suk^61^. All the three have different processing conditions and manufacturing methods. Overall, in this work, optimized working parameters were found (frequency, power, and strain, heating temperature) besides some important physical properties of the muscle for actuation such as length and pre-tensioning to generate the best performance of the muscle in the robot. The utilization of TCP_FL_ to create a low-cost actuation mechanism with the addition of silicone and thin spring steel giving the robot a vertical swimming speed of 7.3 mm/s. When normalized with the bell diameter (150 mm), the speed is 0.05 body length per second.

## Conclusions

In this paper, we studied the performance of a novel method of locomotion underwater with the help of twisted and coiled polymer (TCP_FL_) actuators by getting the contraction–expansion movement of the jellyfish-inspired robot. This robot can be used in expanding the capability of operations and exploration of inaccessible areas in underwater environments. A swimming robot prototype was designed, fabricated, assembled, tested in laboratory environments; and flow simulation was conducted to measure swimming speed for varying input currents, changes in wake flow, actuation displacement and thrust generation. The experimental result of the robot revealed its useful potential of achieving motion without the usage of motors/pumps but only by soft actuators like TCP_FL_ and only few simple components which paves the way for developing similar low-cost underwater swimming robots or soft swarm systems. This will help in leveraging simple, easily manufacturable, bioinspired design and functionality adapted from swimming of a real jellyfish. Simplistic modeling of artificial muscles was presented, and it was effective in predicting the time domain strain response.

In future, to make the manufacturing process more automated, we will work on 3D printing the silicone bell rather than casting it following our recent work and setup^62^, while embedding the spring steels during the 3D printing process. To improve the actuation performance, we will be utilizing mandrel coiled TCP_FL_ actuators or Flexinol NiTi springs^18^, rather than the self-coiled TCP_FL_ actuators used in this paper, as the mandrel-coiled actuators and Flexinol NiTi springs provide larger displacement. The predictive model demonstrated can be used to optimize the amount of input current provided to the TCP_FL_ actuator to give the required amount of actuation displacement needed for the requirements of a particular application. The power consumption of the three TCP_FL_ actuators integrated in the robot is high, which is the result of the actuators using high voltage for their operation. Hence, to make the robot untethered in future, we can connect boost converters with a battery as shown by Hamidi et al.^49^ or apply nanomaterials like graphene^61^ to get better performance from the muscles. This shows that TCP_FL_ actuators can be used to actuate the jellyfish-inspired robot in an untethered field application.

## Methods

### Fabrication of TCP_FL_ muscles

Twisted and coiled polymer fishing line (TCP_FL_) muscles are made by twisting and coiling a nylon 6,6 fiber. The fishing line monofilament after twisting and coiling can provide contraction and expansion movements based on variations in temperature. We followed a similar fabrication process as explained in our previous work^20^. The power source used is DC power supply by BK Precision, model number—9116/B with output rating 0 to 150 V and 0 to 30A. The fabrication methodology (Fig. S2) is explained in detail in the Methods section of the Supplementary File.

### Isotonic testing of TCP_FL_ in an underwater environment

Studying the actuator under various environmental conditions is critical in understanding the performance of such actuator before deploying it in the desired robotic system. An isotonic test consists of testing the actuator under constant stress, varying input power, and varying frequency conditions. A square wave input current was applied to the muscle while data such as strain, temperature, voltage, and current with respect to time is collected. In this case, a square wave input power was used for all tests. The set up included data acquisition sensors such as NI DAQ 9219, Keyence laser displacement sensor, thermocouples, LabVIEW program, power supply, and calibrated weights. The test was conducted at constant input current conditions (0.45A-0.75A) at a frequency of 0.33 Hz and 16.7% duty cycle (5 s heating and 25 s cooling).

### Characterization setup for wake flow dynamics

To study the wake flow dynamics of Jelly-Z for varying actuation displacements with varying input currents (1.8A, 2.0A, 2.2A), experiments were conducted in a recirculation water channel with a section size of 2000 × 200 × 200 mm (length x height x width). The schematic diagram of the experimental set up is shown in Fig. 5 (a). The bioinspired jellyfish prototype was submerged in still water with the robot bell’s lower edge line at 80 mm from water channel bed.

The wake flow dynamics of Jelly-Z were characterized by a planar PIV from TSI for chosen cases of input currents from 1.8A to 2.2A. We use a high-speed 4 MPixel (resolution—2560 × 1600) phantom VEO440 camera to create a field which provides a reaction force as well as a high-pressure region that drives the whole structure forward. In general, thrust of view (FOV) of 245 mm × 155 mm, which covered the full water depth. The origin of coordinate system was set at the bottom left of the FOV. For each measurement, 4500 image pairs were collected at a frequency of 100 Hz. A 1 mm thick 30 mJ/pulse laser was applied to illuminate the FOV, the water channel was seeded with 14 μm diameter silver-coated hollow glass beads with a density of 1.02 g/cm^-3^. For processing the PIV data, an interrogation window with 32 × 32 pixels and 50% overlap was applied with the final vector grid size of δx = δy = 1.78 mm. To identify the seeding particle, the uncertainty of Gaussian fit is around 0.1 pixel, which induces an uncertainty around 1.25% with a bulk particle displacement of 8 pixels between 2 successive images.

### Simplified fluid–structure simulation setup

The bell was designed in SolidWorks, while its overall geometry was reduced to simplify the computational cost by only using a 2D segment of the bell. A cuboid shaped domain was generated around the bell geometry (430 × 560 × 640 mm) as presented in Fig. S1a. The boundaries of the generated domain were wide enough to allow the free flow around the jellyfish model without any obstructions. The simplified fluid–structure simulation was created using ANSYS 2021 R1. We followed steps similar to the ones presented in Almubarak et al.^48^. The simulation is conducted in two steps, structural (step 1) and fluid simulations (step 2). First, the structural simulation which focuses on the jellyfish bell motion (contraction and expansion). The structural simulation applied a displacement boundary condition at the tip of the bell segments, the displacement and time were extracted experimentally from video recording (Fig. S1b,c). The top center of the jellyfish structure was set as a fixed boundary condition. All the faces were assigned with a solid–fluid interaction boundary condition since they will be in contact with the fluid domain.

To reduce the computational cost and time, the bell geometry was simplified to 2 opposite symmetric bells. The material used for the jellyfish was a custom hyper elastic material (EcoFlex00-10) defined by using the Mooney-Rivlin 5 parameter as shown in Table 3.

**Table 3:** Material properties for custom hyperplastic silicone (EcoFlex 00–10)^63^.

Mooney Rivlin 5 parameter	Value
C_10_(Pa)	12,930
C_01_(Pa)	2775.8
C_20_(Pa)	499.19
C_11_(Pa)	− 31.463
C_02_ (Pa)	0.38316
Density	1.07 g/cm^3^

Second, the fluid simulation was performed. The instantaneous velocity with respect to time of the Jelly-Z swimming was also extracted experimentally from video recording. The fluid domain was set with an inlet at the top, outlet at the bottom, and wall boundary conditions with zero shear through the sides of the domain (Fig. S1a). The pressure across the domain is assumed to be 0. A Boolean is implemented to subtract the solid bell from the fluid domain, the solid part retained, and a solid–fluid interaction was set on the surface of the bell. The fluid and solid bodies were meshed separately. After that, a system coupling is created between the first (structural) and the second (fluid) set up for the shared boundary between the jellyfish structure and the surrounding fluid with data transfer. A detailed fluid–structure feedback simulation procedure is presented in the Supplementary Material for further description.

## Supplementary Information

Supplementary Video 1.

Supplementary Legends.

Supplementary Information.

## Data Availability

All data needed to evaluate the conclusions in the paper are present in the paper or the Supplementary Materials.
