# Supplementary material for: Jelly-Z: swimming performance and analysis of twisted and coiled polymer (TCP) actuated jellyfish soft robot
Source: Sci Rep. 2023 Jul 8;13:11086. doi: 10.1038/s41598-023-37611-1 (PMC10329702; doi:10.1038/s41598-023-37611-1)
Supplement: Supplementary file 2 — Supplementary Legends. [file 41598_2023_37611_MOESM2_ESM.docx]

**Legend – for the Movie**

**Movie S1.** Jelly-Z Overview: The structure of the robot, fabrication of TCP_FL_ actuators, swimming tests at different input currents, wake flow PIV tests and FSI simulation <https://youtu.be/WGPiZbDxWfQ> ( also available in Private mode)
