# Supplementary material for: Jelly-Z: swimming performance and analysis of twisted and coiled polymer (TCP) actuated jellyfish soft robot
Source: Sci Rep. 2023 Jul 8;13:11086. doi: 10.1038/s41598-023-37611-1 (PMC10329702; doi:10.1038/s41598-023-37611-1)
Supplement: Supplementary file 3 — Supplementary Information. [file 41598_2023_37611_MOESM3_ESM.pdf]

Supplementary material

Title: Jelly-Z: Swimming performance and analysis of twisted and coiled polymer (TCP) actuated jellyfish soft robot

Methods

Fluid simulation procedure.

The fluid structure feedback simulation set up was conducted by coupling both the structural domain with the fluid domain to be solved in parallel. Fig.S1(a) shows an overview schematic of the computational domain which includes the structural robot geometry in the fluid domain. The structural boundary conditions and values applied are shown in Fig.S1 (b &c).

In this setup, we used Pressure Based solver, velocity formulation as Absolute method, and the time case as Transient. We did not consider gravity in this simulation. Table S2 explains the options considered in the dynamic mesh setting and Table S3 provides the simulation procedures used in the fluent set up.

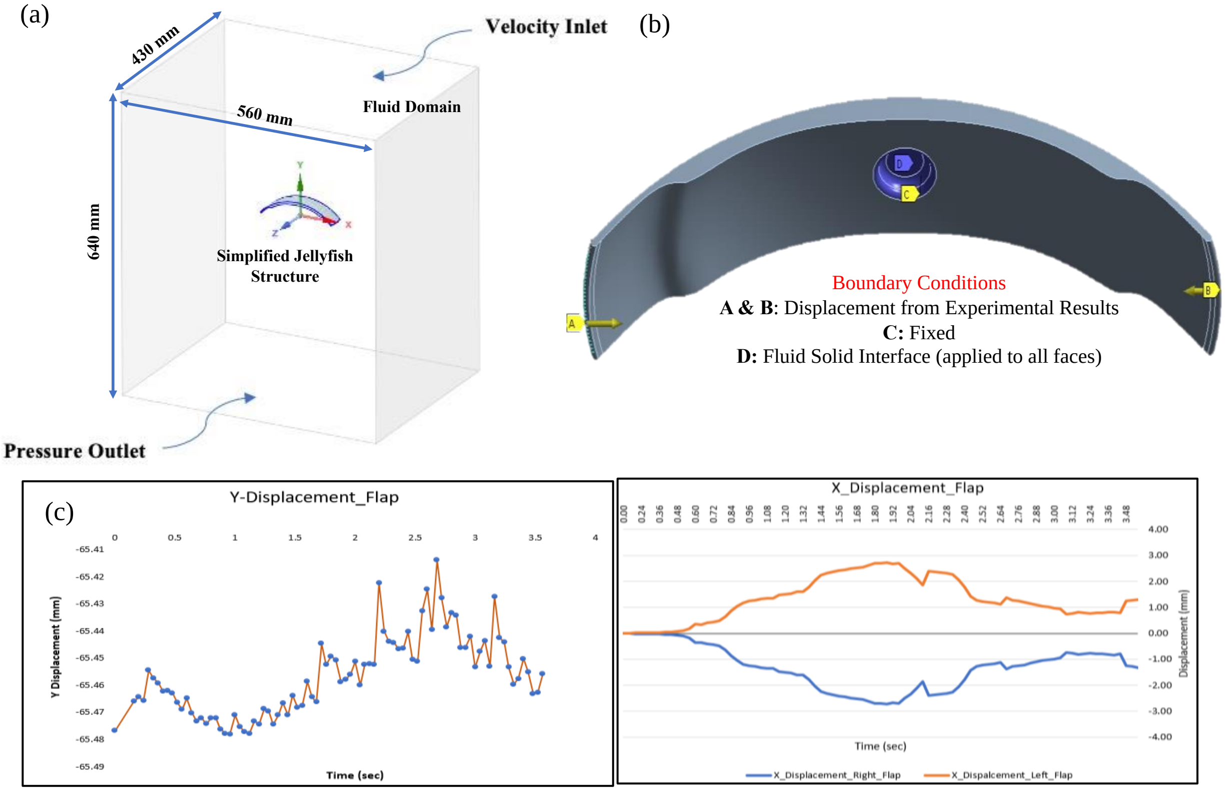

Fig S1: Fluid structure feedback simulation set up (a) Computational domain. (b) Boundary conditions applied on the structural simulation. (c) Extracted experimental displacement applied for the boundary condition

Fabrication of TCP$_{FL}$ Muscles. First, the fishing line monofilament of diameter 0.8 mm was twisted. Second, the nichrome wire of diameter 0.016 mm was incorporated throughout the length of the twisted monofilament at a speed of 150 rpm. Finally, both the fishing line and the nichrome were coiled. Crimps were added to the ends of the coiled actuator to allow for better electrical connection with the power source. The actuator was annealed in an oven at 180°C for 90 minutes to permanently retain its coiled shape. This annealing process aligns the crystal structure of the nylon, which allows it to stay at the coiled shape. Lastly, the muscle is trained at multiple heating cycles following the protocol presented in Matharu et al.[1] and Almubarak et al.[2].
Table 2 provides the physical and electrical characteristics of the fabricated actuator in water. Typically, , the use of hot and cold water or forced air have been presented in Wu et al.[3]. The benefit of this actuation is that the material cost will be extremely low due to the removal of heater wires and improvement in the actuation speed. When TCPs are operated in hydrothermal, the actuation frequency goes higher, i.e 4.5% contraction at 2 Hz while lifting a 7.2 kg was shown in Haines et al.[4]. There are some disadvantages that may arise, specifically, the addition of equipment such as pumps to achieve the exchange of hot and cold water. Therefore, as a solution we opted to use joule heating for actuation. [2,5,6]

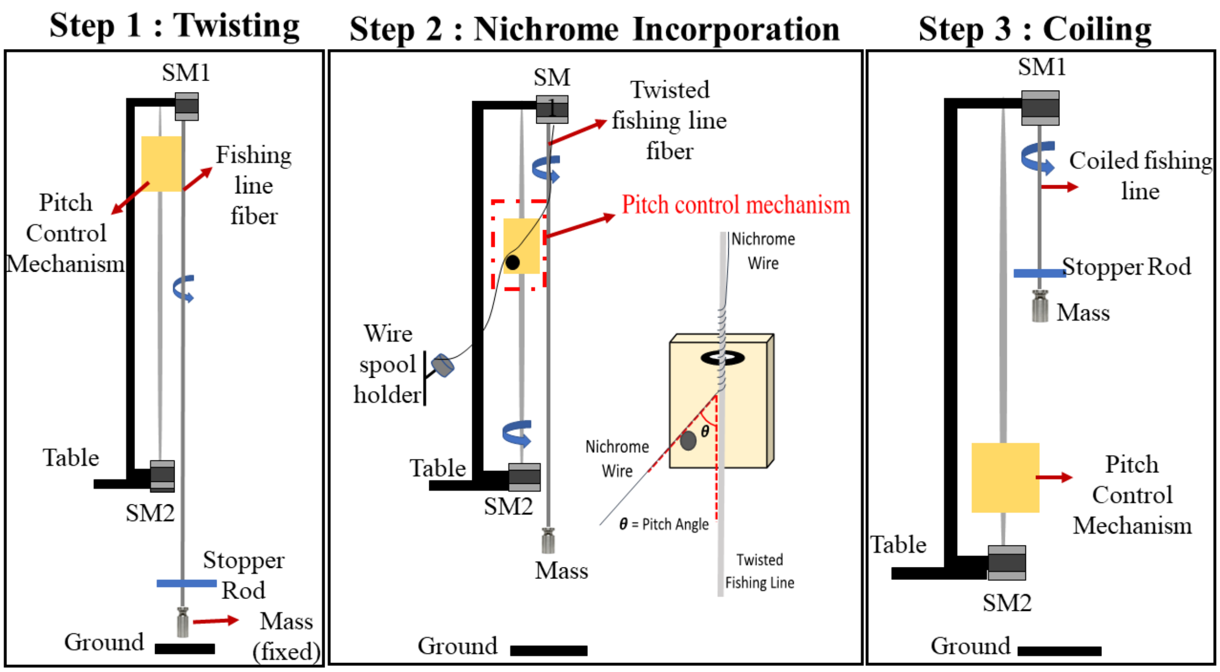

Figure S2: Schematic diagram of the TCP$_{FL}$ muscle fabrication process (left) Twisting process, (middle) Nichrome incorporation process and (right) Coiling process.

Stress – Temperature plots of self-coiled TCP$_{FL}$ Muscles .

From the experimental results (Figure 2, main text), we deduced a linear relationship between the strain and the temperature as presented in the model in equation (4).

$$S(t) = \gamma T(t) + \beta$$

This is reflected in the presented plots in Fig. S3 for both the heating and cooling cycle from the experimental results. As the temperature increases the strain also increases and similarly as it decreases the strain also decreases. In the modeling of temperature and strain , the values of “h” at different input currents are 1529 W/(m^{2}K) for 0.45A, 1580 W/(m^{2}K) for 0.55A, 1603 W/(m^{2}K) for 0.65A and 1550 W/(m^{2}K) for 0.75A. The linear approximation that we considered for theoretical study is based on the heating cycle only and does not consider the cooling cycle. The regression coefficient for linear approximation is as shown in Fig.3(a), which is in the range of 0.92-0.99. This indicated that linear approximation works well for the hearting cycle. Whereas the values of R^2 (Fig. S3 (b)) for the cooling cycle are from 0.67 to 0.87, which means linear approximation is not valid in this region.

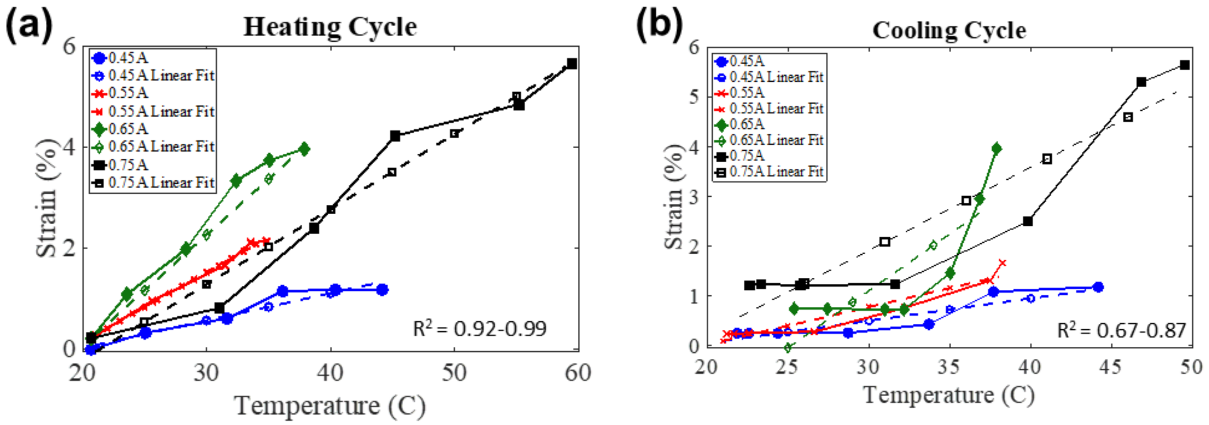

Figure S3. Stress vs strain plot and linear approximation for (a) for heating cycle, (b) for cooling cycle.

Supplementary Tables:

Supplementary Table S1: Comparison of swimming jellyfish robots presented in the literature. This table is modified from Almubarak et al. 2020, Ref [15].

Paper	Actuator	Voltage (V)	Current (A)	Power (W)	Frequency (Hz)	Weight (g)	Bell Diameter (mm)	Vertical Velocity (mm/s)
RoboJelly [7]	SMA wire	---	1.5	~100	0.5	242	164	50
JenniFish [8]	PneuNet	---	---	2.29-5.85	0.8-0.435	380	210	30
DE Jellyfish [9]	Dielectric Elastomer	6000	---	---	---	270	---	10
Modular Jellyfish [10]	SMA wire	---	1	---	0.5	---	216	45
Untethered Jellyfish [11]	Dielectric Elastomer	7000-9000	---	---	1.6	28	156	5-10
Synthetic Jellyfish [12]	Coiled SMA spring	18-19	---	104-115	0.5	---	76	---
JetPRO [13]	Micro DC gear Motor	10	---	---	---	80	30	116
Fludojelly [14]	Pneumatic (Air)	12	---	---	0.8	500	220	160
Kryptojelly [15]	SMA wire	12	30	~360	0.33	650	210	60
Polysaora [16]	6-ply TCP_{Ag}	20	60	~1200	0.25	440	210	5
LM-Jelly	Electromagnetic actuator	7.5	0.62	4.65	0.8	---	---	6
Jelly-Z (Our work in this paper)	TCP_{FL}	56	2.4	~134	0.33	168	150	7.33

Supplementary Table S2: Dynamic Meshing Options Applied

Smoothing		
	Diffusion option	Check box ☑
	Diffusion parameter	2
	Max number of iterations	30
	Remaining options	Default
Remeshing		
	Local cell	Check box ☑
	Local face	Check box ☑
	Region face	Check box ☑
Parameters of Remeshing		
	Mesh scale info	Extracted and entered values in corresponding cells
	Max skewness	0.8
	Max face skewness	0.6
	Size remeshing interval	1
Zones		
	Considered deformation type for the enclosure around jellyfish	Check box ☑
	System coupling	SIX DOF for creating the motion history of the simulation.

Supplementary Table S3: Fluent and solution set up

Physics		
	Viscous	Check box ☑
	Laminar Flow	Check box ☑
Material		
	Water Liquid H_2O	Added
	Air	Deleted
Cell Zones		
	Fluid	Assigned to enclosure
Boundary Setting		
	Inlet	Velocity extracted from experimental video
	Output	Pressure outlet
Solution Method		
	SIMPLIC	Check box ☑
	Spatial discretization gradient	Green Guss node based
	Pressure	2nd order
	Momentum	2nd order upwind
	Transient formulation	2nd order
Definitions		
	Report	Output thrust force
Initialization		
	Hybrid method	Check box ☑
Autosave		
	Iteration saved	Every 4 with time step
	Exported	CDAT for CFD post
Run Calculation		
	Type	Fixed
	Method	User specified
	Number of time steps	356
	Time step size	0.01s
	Maximum iteration per time step	4
	Reporting interval	1

References

1 Matharu, P. S., Ghadge, A. A., Almubarak, Y. & Tadesse, Y. Jelly-Z: Twisted and coiled polymer muscle actuated jellyfish robot for environmental monitoring. *ACTA IMEKO* **11**, 1-7 (2022).

2 Almubarak, Y., Schmutz, M., Perez, M., Shah, S. & Tadesse, Y. Kraken: A wirelessly controlled octopus-like hybrid robot utilizing stepper motors and fishing line artificial muscle for grasping underwater. *International Journal of Intelligent Robotics and Applications*, 1-21 (2022).

3 Wu, L. *et al.* in *SPIE Smart Structures and Materials+ Nondestructive Evaluation and Health Monitoring* (2015).

4 Haines, C. S. *et al.* Artificial muscles from fishing line and sewing thread. *science* **343**, 868-872 (2014).

5 Hamidi, A., Almubarak, Y. & Tadesse, Y. Multidirectional 3D-printed functionally graded modular joint actuated by TCP FL muscles for soft robots. *Bio-Design and Manufacturing* **2**, 256-268 (2019).

6 Wu, L., Chauhan, I. & Tadesse, Y. A novel soft actuator for the musculoskeletal system. *Advanced Materials Technologies* **3**, 1700359 (2018).

7 Villanueva, A., Smith, C. & Priya, S. A biomimetic robotic jellyfish (Robojelly) actuated by shape memory alloy composite actuators. *Bioinspir Biomim* **6**, 036004 (2011). https://doi.org:10.1088/1748-3182/6/3/036004

8 Frame, J., Lopez, N., Curet, O. & Engeberg, E. D. Thrust force characterization of free-swimming soft robotic jellyfish. *Bioinspiration & biomimetics* **13**, 64001-64001 (2018).

9 Godaba, H., Li, J., Wang, Y. & Zhu, J. A Soft Jellyfish Robot Driven by a Dielectric Elastomer Actuator. *IEEE Robotics and Automation Letters* **1**, 624-631 (2016). https://doi.org:10.1109/LRA.2016.2522498

10 Zhou, Y. *et al. A novel biomimetic jellyfish robot based on a soft and smart modular structure (SMS).* (2016).

11 Cheng, T. *et al.* Untethered soft robotic jellyfish. *Smart Materials and Structures* **28** (2018). https://doi.org:10.1088/1361-665X/aaed4f

12 Kazemi-lari, M., Dostine, A., Zhang, J., Wineman, A. & Shaw, J. *Robotic jellyfish actuated with a shape memory alloy spring*. (2019).

13 Marut, K., Stewart, C., Michael, T., Villanueva, A. & Priya, S. A jellyfish-inspired jet propulsion robot actuated by an iris mechanism. *Smart materials and structures* **22**, 094021 (2013).

14 Joshi, A., Kulkarni, A. & Tadesse, Y. FludoJelly: Experimental study on jellyfish-like soft robot enabled by soft pneumatic composite (SPC). *Robotics* **8** (2019). https://doi.org:10.3390/robotics8030056

15 Almubarak, Y., Punnoose, M., Maly, N. X., Hamidi, A. & Tadesse, Y. KryptoJelly: a jellyfish robot with confined, adjustable pre-stress, and easily replaceable shape memory alloy NiTi actuators. *Smart Materials and Structures* **29**, 075011 (2020).

16 Hamidi, A., Almubarak, Y., Rupawat, Y., Warren, J. & Tadesse, Y. Poly-saora robotic jellyfish: Swimming underwater by twisted and coiled polymer actuators. *Smart Materials and Structures* **29** (2020). https://doi.org:10.1088/1361-665X/ab7738
